# Editorial

**Published:** 1913-04

**Authors:** 


### EDITORIAL

The Business Office of the *Journal-Record* is 23 West Alabama Street.

The Editorial Office is 1014-15 Atlanta National Bank Building.

Address all Business Communications to *Journal-Record of Medicine,* 23 West Alabama Street.

Make remittances payable to *The Journal-Record of Medicine.*

On matters pertaining to the Editorial and Original Communications, address Edgar G. Ballenger, M. D., Atlanta, Ga.

Reprints of Original Articles will be furnished at cost price. Requests for the same should always be made in THE MANUSCRIPT.

We will present, postpaid, on request to each contributor of an original article, twenty (20) marked copies of *The Journal-Record of Medicine* containing such article.

### AUTOSEROTHERAPY

In 1891 Gilbert began the treatment of tuberculosis pleurisy with subcutaneous injections of small amounts of the effusion drawn from the pleural cavity with an aspirating syringe. He found this to be a very satisfactory treatment, but the method was very slow in gaining recognition. During recent years however, reports from European clinics have shown that serum therapy is of value and Fishburg, in the Journal of the Amreican Medical Association, March 29, 1913, urges the more general adoption of this form of therapy. Eisner's animal experiments show that not only does the withdrawal of blind stimulate absorption but that the subcutaneous injection of the fluid is responsible for the further absorption of the excidate in autoserotherapy. During the past four years Fishberg has treated twelve patients with pleuritic effusion in this manner and while he says he cannot regard it as specific, because it has not cured every patient, he asserts that it has sufficient merit to warrant its more general adoption. The technic is that of exploratory puncture, with observance of all antiseptic precautions. From 2 to 5 c.c. of the fluid is immediately reinjected into the cellular tissue of the abdomin or elsewhere. In suitable cases improvement soon follows The treatment may have to be repeated several times. Nine of Fishberg's twelve cases were distinctly benefitted; in one the result was doubtful and in two it failed completely.

The New York Medical Journal of March the 29th, the following abstract from the Review de Medicine:

Vitry and Seary present the history of an undoubted case of cirrhosis, with diminished size of the liver, splenic enlargement, prominent collateral circulation, and positive methylene blue test, in which autoserotherapy and other measures led to rapid absorption of an ascitic accumulation of six months' standing. The patient was put to bed and given three pints of milk a day. Upon administering one gramme of theobromine the elimination of urine rose silghtly. A week later, subcutaneous injections of ten c.c. of asctic fluid were started, and therafter continued on alternate days. On the third day a free flow of urine set in, with the result that eleven days later the ascites had disappeared and autoserotherapy was necessarily discontinued. The urine during this period showed both an absolute and a relative increase in sodium chloride—a fact apparently of considerable significance in relation to the favorable results obtained. The urinary acidity was simultaneously lessened, owing to the elimination of the lkaline ascitic fluid. The patient was subsequently given saltfree food, then placed on a careful chloride-containing diet, with the result that ascites and the symptoms due to poral congestion have not returned.

---

### ORGANIZATION OF GEORGIA SURGEONS' CLUB

During the session of the Medical Association of Georgia in Savannah, April 17th, 1913, the Georgia Surgeons' Club was organized, this to be modeled somewhat after the organization which has attained so much success, organized by Dr. Franklin Martin of Chicago and which has proven to be one of the most interesting methods of advancing surgical principles and improving the surgery throughout the country. This of course, is to be on a very much smaller scale and it is intendec to hold a clinic for one or two days once each year in such cities in the State as have hospital facilities.

In addition to this, it is the hope that such members as desire may be enabled to procure rates on the railroads when they wish to visit some clinic at a distance.

The surgical work and technique of our own State will compare most favorable with that of any other state in this country and if the various surgeons may be enabled to become familiar with the work done by their neighbors, he may often learn more than by taking an expensive and tedious trip. Besides this, it is the intention of this organization to have night sessions and have some surgeon of eminence to address the meeting on original work.

Georgia should keep abreast of all the States in the United States in its surgical progress as it now has the loftiest position in giving to the world so many men who have done the original things that are valuable in surgery.

The following officers were elected at this meeting:

Dr. E. C. Davis, Atlanta, President; Dr. T. J. McArthur, Cordele, Vice-President; Dr. R. M. Harbin, Secretary-Treasurer.

The President appointed the following executive committee to arrange for the next meeting and to perfect plans for the organization:

Dr. W. S. Goldsmith, Atlanta; Dr. G. R. White, Savannah; Dr. W. W. Battey, Augusta.

All members of the medical profession, actively engaged in surgical work, who may desire to identify themselves with the organization, should communicate at once with the Secretary, Dr. R. M. Harbin, Rome, Georgia.

The first meeting will be held in Atlanta either in October or November, 1913.

---

Don't urge the radical operation for frontal sinusitis unless the symptoms are severe or conservative efforts have failed; the operation is disfiguring and the results are not always satisfactory.—*American Journal of Surgery.*

